# Chronic kidney disease and fragility fracture

**DOI:** 10.1007/s10157-016-1368-3

**Published:** 2016-12-23

**Authors:** Junichiro James Kazama

**Affiliations:** Department of Nephrology and Hypertension, Fukushima Medical University, 1 Hikarigaoka, Fukushima, Fukushima 960-1295 Japan

**Keywords:** Fragility fracture, Osteoporosis, Chronic kidney disease-related bone and mineral disease (CKD-MBD), Fracture liaison

## Abstract

Osteoporosis is defined simply as “a skeletal disorder characterized by compromised bone strength predisposing to an increased risk of fracture. Thus, any bone lesion that causes fragility fracture is osteoporosis, which has quite heterogeneous backgrounds. Chronic kidney disease-related bone and mineral disease (CKD-MBD) is defined as “a systemic disorder of mineral and bone metabolism due to CKD, which is manifested by abnormalities in bone and mineral metabolism and/or extra-skeletal calcification”. Although CKD-MBD is one of the possible causes of osteoporosis, we do not have evidences that CKD-MBD is the only or crucial determinant of bone mechanical strength in CKD patients. The risk of hip fracture is considerably high in CKD patients. Drugs that intervene in systemic mineral metabolism, indeed, lead to the improvement on bone histology in CKD patients. However, it remains unclear whether the intervention in systemic mineral metabolism also improves bone strength, today. Thus, the use of drugs that directly act on bone and the introduction of fracture liaison concept are promising strategies for fragility fracture prevention among CKD patients, as well as treatment for CKD-MBD.

## Introduction

Various pathological conditions are found in bone among patients with chronic kidney disease (CKD), and the pathophysiological mechanisms that underlie these lesions are also complicated. Here, I briefly review the present condition of fragility bone fracture and its treatment in CKD patients.

## Osteoporosis and CKD-MBD

Osteoporosis is defined by the World Health Organization as “a skeletal disorder characterized by compromised bone strength predisposing to an increased risk of fracture” [1]. This definition omits the following, which had been included in the previous definition [2]; “low bone mass” and “microarchitectural deterioration of bone tissue”. The revision clearly indicates that the latter conditions are no longer a requirement or a sufficient condition of osteoporosis. In other words, any bone condition that causes fragility fracture is now considered as osteoporosis. However, the disease definition is frequently misunderstood, because bone mass measurement is still the standard method to diagnose osteoporosis. Although osteoporosis is a disease characterized by compromised bone strength, there is no practical tool to monitor bone mechanical strength at bedside, today. Bone mass is, indeed, a strong determinant of bone mechanical strength [3]; bone mass is used as a diagnosing tool for osteoporosis under the premise that extremely low bone mass could be regarded as a sufficient condition of compromised bone strength.

Since low bone mass is neither a requirement nor a sufficient condition of compromised bone strength, a current diagnosis of osteoporosis is quite inaccurate, which could lead to both false-negative and false-positive cases. Yet, we do not have any other practical tools that indicate bone strength, with the exception of a patient’s medical history. Thus, bone mass measurement is considered the most powerful tool available today to diagnose osteoporosis.

However, bone mass is not the only determining factor of bone mechanical strength. Factors other than bone mass that determine bone mechanical strength are generally considered aspects of “bone quality” [4]. It has often been said that “bone mechanical strength is predominantly prescribed by bone mass, and bone quality contributes”, which seems to be true in most of the cases, but not all the cases. For example, bone mineral density is generally low in elementally school children, and although they fall quite often, they seldom suffer from fragility fractures. Thus, bone quality is sometimes likely to be a more important factor than bone mass for preventing fragility fracture, at least in some patient populations. At present, the ratio of importance of bone mass and bone quality is not clear.

A primary reason for this is that it is difficult to define bone mechanical strength. Because the risk of fragility fracture incidence is also dependent on the risk of fall, it does not strictly represent bone mechanical strength. In ex vivo destruction studies using extracted bone samples, different results will be obtained based on the direction or moment of force applied to the samples. Moreover, bone hardness and viscoelastic properties are different characteristics that contribute independently to the resistance against destruction [5]. Thus, osteoporosis is a heterogeneous disease condition in that many factors contribute to bone fragility with different proportions in each case. The disease background is also quite heterogeneous.

Chronic kidney disease-related bone and mineral disease (CKD-MBD) is defined by the Kidney Disease: Improving Global Outcomes as “a systemic disorder of mineral and bone metabolism due to CKD, which is manifested by abnormalities in bone and mineral metabolism and/or extra-skeletal calcification” [6]. CKD-MBD is a disease consisting of three inter-related components: abnormal laboratory examination results including parathyroid dysfunctions, bone metabolic abnormalities and abnormal soft tissue calcification including vascular calcification. At one time, the term “renal osteodystrophy (ROD)” indicated a disease condition similar to CKD-MBD, but it is now considered simply a pathomorphological feature that indicates bone lesions associated with CKD-MBD [7].

**Fig. 1:**
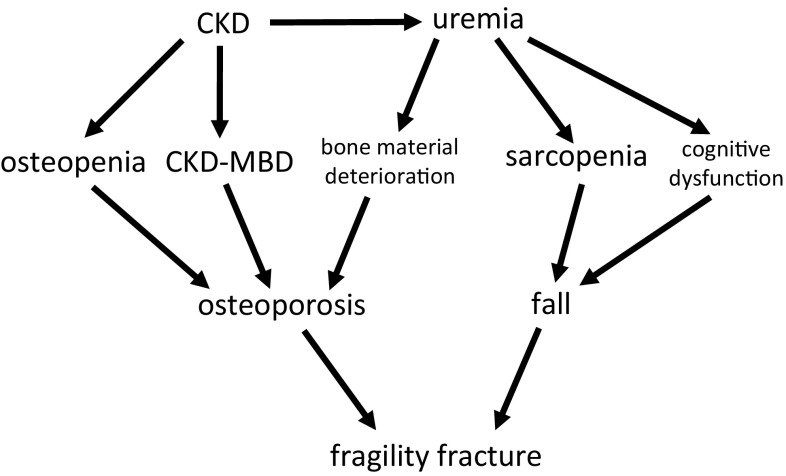
The pathophysiological mechanism of fragility fracture in CKD patients. Direct cause of fragility fracture is osteoporosis. Generally, the major cause of osteoporosis is osteopenia, and osteopenia is common in CKD patients. Uremia is likely to deteriorate bone material properties. According to the disease concept, bone fragility is not the requirement of CKD-MBD. However, CKD-MBD has a potential to cause osteoporosis. The frequency of the fall is another major risk of fragility fracture, and uremia also increases the risk of fall

According to the definition of CKD-MBD, bone lesions caused by abnormalities in systemic mineral metabolism associated with CKD are partial features of CKD-MBD, but those lesions do not have to be accompanied by the deterioration of bone mechanical strength. Bone disease with deteriorated mechanical strength is osteoporosis, and CKD-MBD is one of the possible causative backgrounds of osteoporosis (Fig. 1). However, even in those cases in which CKD-MBD has some roles in the deterioration of bone strength, other factors could also simultaneously contribute to the development of osteoporosis, as osteoporosis has quite heterogeneous backgrounds. We previously advocated a disease concept termed “uremic osteoporosis” that is caused by uremic toxins but not abnormal mineral metabolism [8]. Although many studies are required before understanding this disease condition in detail, it is certain that there are various backgrounds lying behind the bone fragility among patients with CKD. It is, thus, a leap of logic to conclude that CKD-MBD is the only or crucial determinant of bone mechanical strength in CKD patients.

## Bone fracture in CKD patients

It has been confirmed that the hip fracture (HFx) risk is considerably high in dialysis patients in many countries [9–11] including Japan [12]. The risk is 4–13 times higher in dialysis patients compared to the general population. The risk tends to be higher in high-latitude regions in the United States [13]. In Japan, the risk is higher in western prefectures [14], indicating that sunlight has little to do with the HFx risk. Although many investigators consider that the risk is also higher in predialysis CKD patients [15, 16], an opposing view has been reported [17]. A few reports contended that the risk of spinal compression fracture is also high in dialysis patients [18].

Falls are common in especially elder CKD patients [19], which is very likely to be one of the major causes of elevated HFx risk. Advanced muscle weakness [20], frailty [21], and deteriorated cognition [22] are potential contributors to elevated risk of falling among CKD patients. The survival prognosis generally becomes poorer in patients who have incurred a HFx, and this trend is more evident in dialysis patients [23, 24]. Japanese dialysis patients demonstrated relatively better life prognosis after HFx [25]. However, FRAX, a tool to predict future fracture risk, can predict the risk of mortality even in Japan [26].

It is difficult to know whether being at a high risk of falling is the major cause of the elevated HFx risk in dialysis patients. The majority of clinicians and clinical researchers assume that bone mechanical properties are also deteriorated among dialysis patients. If this assumption is true, many dialysis patients are suffering from osteoporosis.

## What happens in bone when kidney is injured?

The kidney is a pivotal organ for systemic mineral metabolism. When a patient’s kidney is injured, the systemic mineral metabolism is affected and the function of bone, which is another pivotal organ for systemic mineral metabolism, is altered through the collapsed feedback system. This is the general idea regarding the development of bone lesions in CKD-MBD.

Among the many humoral factors affecting bone metabolism in disease states, the largest influence is brought by parathyroid hormone (PTH). PTH is secreted by parathyroid gland, which is hyperactivated under CKD conditions. PTH promotes the activity of bone cells, namely osteoblasts, osteocytes and osteoclasts, at microscopic levels. In macroscopic levels, PTH causes cortical thinning and porosis [27] that could result in increased bone fragility. In fact, elevated serum level of alkaline phosphatase, a likely marker for accelerated bone metabolic activity, is reported to be associated with a higher risk of HFx incidence [28]. However, a controversy still remains on the relationship between parathyroid function and structure-related bone strength [29, 30]. One possible explanation for this issue is that extremely high plasma level of PTH is required to cause evident cortical thinning in dialysis patients [31], because skeletal resistance to PTH stimuli is increased in uremic condition [32–34].

**Fig. 2:**
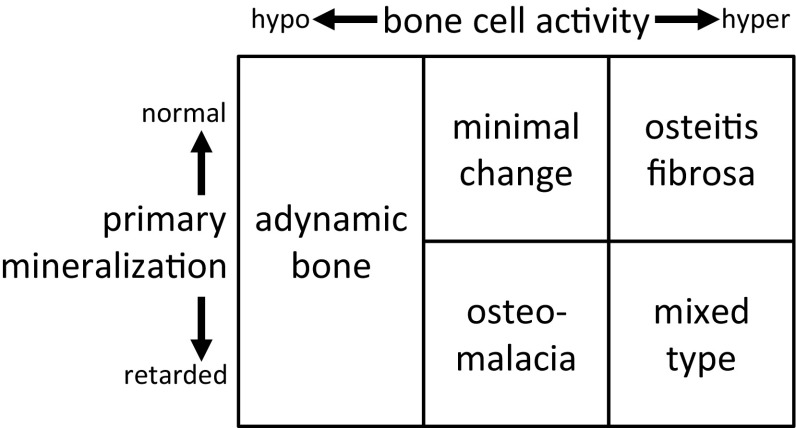
Classic ROD classification. Histological findings of bone samples obtained by bone biopsy are classified into 5 categories by two assessing axes; bone cell activities and primary mineralization speed. This classification is suitable to evaluate bone metabolic condition in CKD patients because CKD patients demonstrate broad spectrums in these two assessing axes. The logic of this classification is obviously clearer than that of the turnover–mineralization–volume classification, which was advocated subsequently to this classification. However, the need of multifaceted information is recognized for an assessment of bone, and bone histology alone is not capable to meet the need, today. Histological findings obtained from bone sections, in fact, give no information about bone biochemical or mechanical properties, and only limited information about the structural properties. Thus, bone biopsy is not almighty. Considering it as the “gold standard” is an overvaluation

Bone condition that has shown an extremely low frequency of bone remodeling, which is called “adynamic bone” [35] is also observed in dialysis patients, presumably due to increased skeletal resistance. Investigators have speculated that in the adynamic bone condition, the bone mechanical strength could be deteriorated through accumulated microdamages [36]. However, the relationship between bone fragility and each category of the classic ROD classification (Fig. 2) remains unknown. This classification is dependent on bone metabolism but not bone mechanical properties [37].

Treatment for hyperparathyroidism in CKD patients has been greatly improved [38]. Recent clinical studies have obtained controversial results regarding the relationship between PTH and HFx risk [39–42]. The decreased incidence of severe secondary hyperparathyroidism due to the development of medical and surgical treatment seems to have contributed to these unclear results. Moreover, osteomalacia caused by the depositions of aluminum or iron on a skeletal calcified front has been nearly eliminated [43]. Thus, the contribution of CKD-MBD to the development of bone fragility can be expected to have decreased today compared to that in the 1980s. Yet, the HFx risk in dialysis patients has remained extremely high, today. We must, therefore, consider the effect of factors other than CKD-MBD that may promote bone fragility in CKD patients.

Bone mass is regarded as the most important and crucial factor in the prediction of bone mechanical properties in individuals with primary osteoporosis. Bone mass seems to be a major determinant of bone strength among dialysis patients also. In CKD patients, the bone mass starts to decrease at the early predialysis period [44]. Abnormal vitamin D metabolism, hypoproteinemia, treatments for kidney disease, and parathyroid dysfunction are assumed to be listed as possible causative factors for low bone mass in CKD patients. By the time of dialysis initiation, an individual’s bone mineral density is generally low, but the bone mass may not decrease as fast as it did in the predialysis period. The promotion of osteoblast apoptosis was shown to be a likely pathophysiological mechanism through which severe hyperparathyroidism causes extensive bone loss in dialysis patients [45], in addition to the promotion of osteoclastic bone resorption. The clinical significance of bone mass measurement using dual-energy X-ray absorptiometry was not confirmed among dialysis patients [46]. However, recent studies have revealed that lower bone mass is associated with a higher fracture risk even in dialysis patients [47, 48].

Many clinicians/clinical investigators have an impression that bone quality in dialysis patients is deteriorated, although no clear evidence exists on this issue. Bone structural properties that could decrease bone mechanical strength are changed in dialysis patients [49]. In experimental animal models, kidney injury caused the deterioration of bone material properties [50]. In those animals, a viscoelastic property of extracted long bones was deteriorated, and both the modification of type-I collagen with advanced glycation end-products (AGE) and bone apatite disorientation were significantly associated with the deterioration [51]. The deterioration in bone material properties was specifically observed in the uremic condition, but it was not associated with abnormal mineral metabolism. Therefore, strictly speaking, these bone abnormalities cannot be regarded as a feature of CKD-MBD [8]. The AGE-modified type-I collagen was also detected in bone samples obtained from dialysis patients [52].

## Fragility fracture prevention in CKD patients

The original reason for using anti-osteoporotic agents was to strengthen bone mechanical properties, leading to a reduction in the fracture risk. However, most of the existing anti-osteoporotic agents directly act on bone to increase bone mass, and therefore, those drugs are referred to as direct bone metabolic modulators in this manuscript. Bone mass is generally low in CKD patients, and low bone mass increases fracture risk. The use of direct bone metabolic modulators that increase bone mass would, thus, seem to have certain benefit for CKD patients. Although low bone mass is neither a requirement nor a sufficient condition of compromised bone strength, it is certain that increasing bone mass strengthens the bone mechanical property, at least to some extent.

There is a significant limitation regarding the use of direct bone metabolic modulators in patients with kidney dysfunction. Bisphosphonate, which is most commonly applied for treatment against primary osteoporosis, is eliminated through urinary extraction, and there is a risk of bisphosphonate accumulating in bone when it is used for dialysis patients [53]. Accumulated bisphosphonate in bone has the potential to reduce viscoelastic properties [54], which could increase the risk of atypical femoral fracture [55–57]. Because bisphosphonate is highly selectively distributed to bone tissue, it is difficult to monitor its accumulation clinically.

Unlike bisphosphonate, the human monoclonal antibody denosumab does not accumulate even in CKD patients. However, its hypocalcemic action tends to be amplified in CKD patients [58]. It must be in mind that hypocalcemia in dialysis patients directly induces an aggravation of hyperparathyroidism [59]. Although teriparatide is, indeed, effective in some dialysis patients, its vasodilative action often compels a discontinuation of the treatment because it can induce severe hypotension. Regarding the clinical effect of selective estrogen receptor modulators in CKD patients, no clear consensus has been established [60].

CKD-MBD is a consequence of abnormal systemic mineral metabolism. Therefore, drugs that intervene in systemic mineral metabolism are frequently used for a CKD-MBD treatment. Vitamin D receptor activator (VDRA) and calcimimetics may provide a decreased fracture risk by suppressing hyperactivated parathyroid function. These systemic mineral metabolism modulators surely lead to a certain improvement in bone histology; in other words, they change bone metabolism. However, the accurate effects of these agents on bone mechanical properties are not yet verified. VDRA also demonstrates a local action on bone. Long-acting VDRA, such as alfacalcidol [61] or eldecalcitol [62] in particular, have the likely potential to prevent fragility fracture through their own direct actions on bone. A study conducted in the United States indicated that the use of cinacalcet hydrochloride decreased the fracture risk among dialysis patients [63] via a yet unverified mechanism. VDRA and cinacalcet may prevent fragility fracture through intervening in vitamin K metabolism [64]. Further studies are required to elucidate whether cinacalcet merely intervened in systemic mineral metabolism or also acted directly on bone to provide the decreased fracture risk.

PTH is a humoral factor that affects systemic mineral metabolism; teriparatide was developed to promote bone formation directly by the intermittent use. Yet, no consensus has been established about the indication of teriparatide therapy among CKD patients. The indication may not be confined to those with PTH levels lower than the target range indicated by clinical practice guidelines, because circulating PTH molecules are unlikely to perform full function in CKD patients [65]. The risk of hospitalization due to any fracture among dialysis patients was approximately 30% lower in the patients treated with an angiotensin-converting-enzyme inhibitor or AT-1 receptor blocker (Renin–Angiotensin–Aldosterone System inhibitor, RAASi) among dialysis patients [66]. Such a phenomenon has not yet been confirmed in a non-CKD population. If bone material properties are truly deteriorated in CKD patients, it is possible that RAASi affect bone material properties by preventing oxidation stress. The serum concentration of bicarbonate ion has a significant relationship with fracture risk [67].

**Fig. 3:**
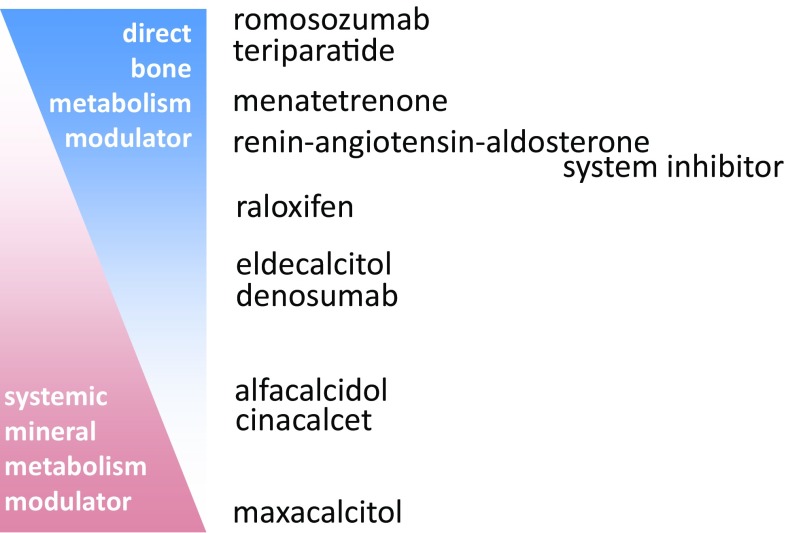
Drugs possibly reduce the risk of fragility fracture among CKD patients

Most of the drugs have more or less both aspects of direct bone metabolism modulator and systemic mineral metabolism modulator (Fig. 3). Although systemic mineral metabolism modulators have been mainly prescribed to CKD patients, the use of direct bone metabolic modulators will be increased in near future from a perspective of fragility fracture prevention.

Most fragility fractures occur due to a fall. The prevention of falls is, thus, another important strategy in fragility fracture prevention, and in fact, it is the most practical tactic at this moment. The so-called fracture liaison concept [68, 69] is now gaining attention as an effective tool to prevent fragility fractures among the elderly. Similar attempts should be made for fracture prevention among CKD patients, especially dialysis patients.

## Conclusion

“Osteoporosis is a more severe form of osteopenia”. “The bone abnormality observed in CKD patients is CKD-MBD, but not osteoporosis”. “Aggressive treatment for CKD-MBD is the major strategy for reducing the fragility fracture risk in CKD patients”. A close examination of these statements suggests the conclusion that the above propositions are not logically true. However, many clinicians and clinical researchers still seem to believe them [70, 71]. Incorrect assumptions hamper the development and spread of useful treatment by providing meaningless research questions and misleading the interpretation of the evidences obtained. We must deal with fragility fracture prevention among CKD patients with a flexible and logical way of thinking.
